# Mechanisms of Electrical Activation and Conduction in the Gastrointestinal System: Lessons from Cardiac Electrophysiology

**DOI:** 10.3389/fphys.2016.00182

**Published:** 2016-05-31

**Authors:** Gary Tse, Eric Tsz Him Lai, Jie Ming Yeo, Vivian Tse, Sunny Hei Wong

**Affiliations:** ^1^Li Ka Shing Faculty of Medicine, School of Biomedical Sciences, University of Hong KongHong Kong, China; ^2^School of Medicine, Imperial College LondonLondon, UK; ^3^Department of Physiology, McGill UniversityMontreal, QC, Canada; ^4^Department of Medicine and Therapeutics, Institute of Digestive Disease, LKS Institute of Health Sciences, Chinese University of Hong KongHong Kong, China

**Keywords:** cardiac electrophysiology, gastrointestinal electrophysiology, electrical excitation, conduction, propagation

## Abstract

The gastrointestinal (GI) tract is an electrically excitable organ system containing multiple cell types, which coordinate electrical activity propagating through this tract. Disruption in its normal electrophysiology is observed in a number of GI motility disorders. However, this is not well characterized and the field of GI electrophysiology is much less developed compared to the cardiac field. The aim of this article is to use the established knowledge of cardiac electrophysiology to shed light on the mechanisms of electrical activation and propagation along the GI tract, and how abnormalities in these processes lead to motility disorders and suggest better treatment options based on this improved understanding. In the first part of the article, the ionic contributions to the generation of GI slow wave and the cardiac action potential (AP) are reviewed. Propagation of these electrical signals can be described by the core conductor theory in both systems. However, specifically for the GI tract, the following unique properties are observed: changes in slow wave frequency along its length, periods of quiescence, synchronization in short distances and desynchronization over long distances. These are best described by a coupled oscillator theory. Other differences include the diminished role of gap junctions in mediating this conduction in the GI tract compared to the heart. The electrophysiology of conditions such as gastroesophageal reflux disease and gastroparesis, and functional problems such as irritable bowel syndrome are discussed in detail, with reference to ion channel abnormalities and potential therapeutic targets. A deeper understanding of the molecular basis and physiological mechanisms underlying GI motility disorders will enable the development of better diagnostic and therapeutic tools and the advancement of this field.

## Introduction

The heart is responsible for delivering oxygenated blood to, and removing deoxygenated blood from, active respiring tissues in the periphery. Its electrical and mechanical activity is tightly regulated and further modulated by neuroendocrine signals. By contrast, in the gastrointestinal (GI) tract, the stomach initiates digestion and delivers gastric contents via the pylorus to the small intestine in a regulated manner. The intestines then further digest and absorb the contents. Both the heart and the gastrointestinal (GI) tract are electrically excitable. Normal mechanical functions of these organ systems depend on the highly coordinated activity of this electrical excitation, whose disruptions can lead to arrhythmias (Tse, 2015, 2016a,b,c; Chen Z. et al., 2016; Choy et al., 2016; Tse et al., 2016a,b,c,e,f,g; Tse and Yan, 2016). Whilst the electrophysiological properties of the heart have been extensively studied, those of the GI tract are relatively less well characterized. This is perhaps because arrhythmias in this system is usually not life-threatening (O'Grady et al., 2014), whereas those in the ventricles can cause sudden cardiac death (Murakoshi and Aonuma, 2013). However, there is increasing evidence that electrophysiological abnormalities play important roles in GI motility disorders such as gastroesophageal reflux disease (Shafik et al., 2005), achalasia (Faussone-Pellegrini and Cortesini, 1985a; Goldblum et al., 1994), Allgrove syndrome (Khelif et al., 2003), gastroparesis (O'Grady et al., 2012), pyloric stenosis (Langer et al., 1995; Vanderwinden et al., 1996), functional dyspepsia (Jung et al., 2012), idiopathic rapid gastric emptying (Bharucha et al., 2011), unexplained nausea and vomiting (Abell et al., 2009), mesenteric ischaemia (Irimia and Wikswo, 2008), functional diarrhea (Dellon and Ringel, 2006), or constipation (Camilleri, 2011), irritable bowel syndrome (Saito et al., 2009), Hirschsprung disease (Yamataka et al., 1995), chronic pseudo-obstruction (Feldstein et al., 2003), slow transit constipation (Lyford et al., 2002), and colonic hypomotility associated with anorectal malformations (Kenny et al., 1998). These conditions cause significant morbidity in the population and it is therefore important to understand their underlying mechanisms for devising effective treatment. A comparison between these systems may provide some insight for the GI electrophysiologists and physicians. Thus, the aim of this article is to examine the conduction pathways, the ionic currents responsible for electrical activation of different cell types, and mechanisms of their propagation in both the heart and the GI tract. This is followed by a discussion on the clinical relevance and molecular targets for future therapy.

## Specialized conduction pathways

**Figure 1:**
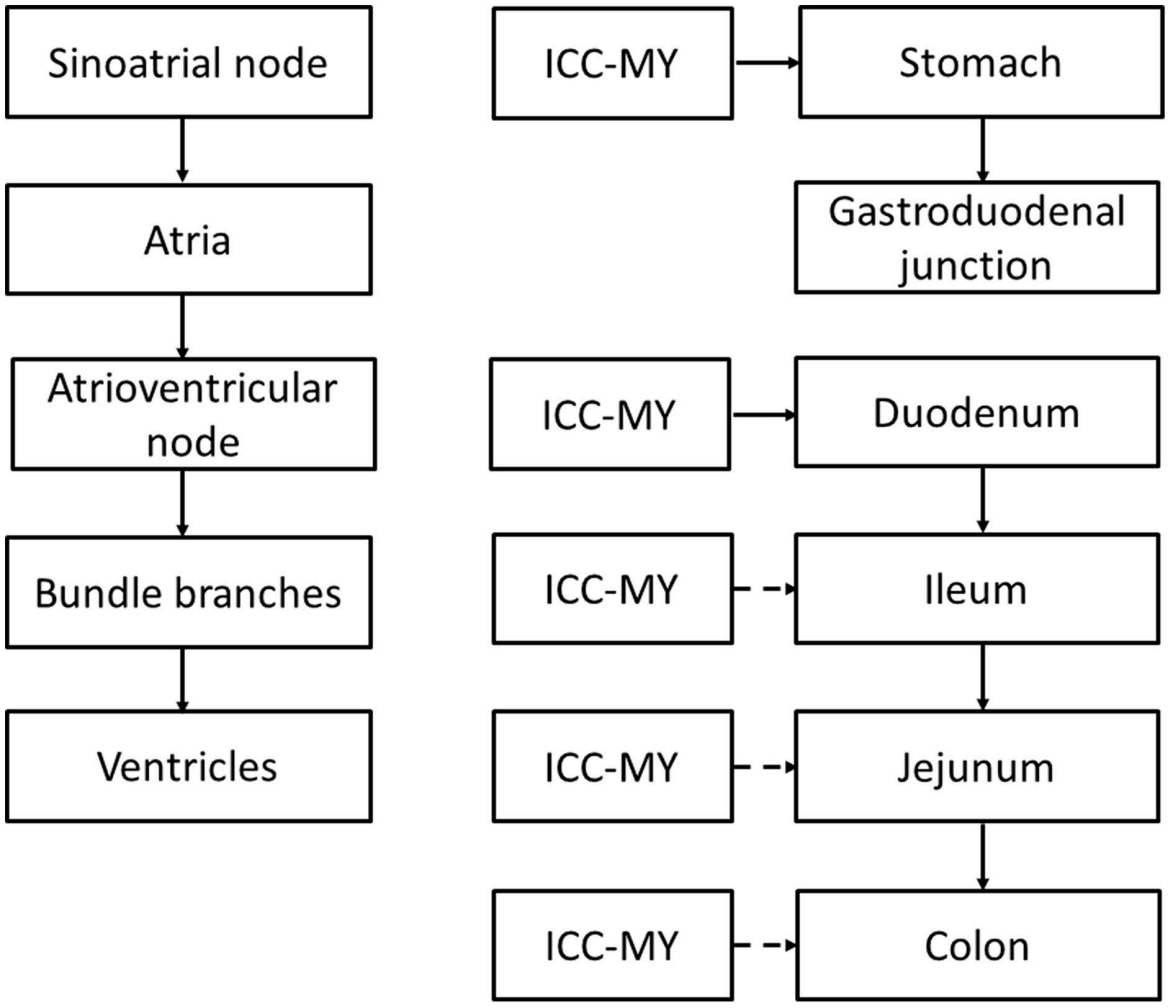
**Comparison between the cardiac and gastrointestinal conduction pathways**. ICC-MY: interstitial cells of Cajal in the myenteric plexus, pacemaker cells of the GI tract. Broken arrows indicate that the ICC is normally reset by the dominant pacemaker upstream that has the highest rate of discharge.

There are specialized conduction pathways responsible for electrical conduction through the heart and the GI tract (Figure 1; Veeraraghavan et al., 2014a). Pacemaker cells are responsible for the spontaneous initiation of electrical activity in both systems. In cardiac tissue, the dominant pacemaker is the sinoatrial node, which is responsible for initiating action potentials (APs) that spread along the cardiac conduction system, reaching all parts of the myocardium. They first spread radially through the right and left atria, converging on the atrioventricular (AV) node, where conduction velocity (CV) is reduced. This brief delay ensures atrial and ventricular systole occurs sequentially. The APs then propagate along the Bundle of His, and then the left and right bundle branches, activating the Purkinje fibers. From there they propagate to the apex and then into the ventricular myocardium. The SA node receives innervation from both the sympathetic and parasympathetic nervous systems, which exert positive and negative chronotropic effects, respectively.

By contrast, the pacemaker region of the gastrointestinal tract is the interstitial cells of Cajal (ICC) of the myenteric plexus (ICC-MY) found in stomach, small intestine and colon, generating slow wave activity that spread into the circular and longitudinal muscle layers (Suzuki et al., 1986; Langton et al., 1989; Ward et al., 1994; Huizinga et al., 1995; Dickens et al., 1999). Interestingly, although ICC have been found in the esophagus, very few of these are associated with the myenteric plexus and therefore they have little role in pacemaker activity (Faussone-Pellegrini and Cortesini, 1985b). Instead, they are of the intramuscular subtype (ICC-IM) in close contact with nerves and smooth muscles. Nevertheless, there is some evidence to suggest that ICC-IM, in conjunction with PDGFRα-positive fibroblast-like cells, together are responsible for electrical and mechanical activation of the esophagus (Chen et al., 2013). In the stomach, ICC-MY is located high up at the greater curvature. The slow waves propagate radially in both the longitudinal and circumferential directions and the main direction of propagation is along the longitudinal direction toward the distal antrum (Lammers et al., 2009). Interestingly, the slow wave does not propagate from the stomach to the small intestine, suggesting either a loss of ICC (Wang et al., 2005) or electrical coupling between these regions (Lammers et al., 1998). The latter is unlikely because conduction of slow waves from the stomach into the pylorus has been observed (Sanders and Vogalis, 1989; Lammers et al., 2000; Wang et al., 2005). Thus, slow wave activity of the stomach and small intestine occurs independently of each other. This is consistent with the predominant role of the pylorus in controlling the flow of contents out of the stomach rather than conducting slow waves to the small intestine. Nevertheless, upon relaxation of the pylorus, there is coordinated activity between the stomach and small intestine, where synchronized propagation of peristaltic activity in the duodenum occur after stomach contractions. This coordination may involve stretch-activated or neural activation mechanisms rather than direct electrical coordination *per se*, as occurs in the heart for atrioventricular delays. Peristalsis in the small intestine propagates from the gastroduodenal junction toward the terminal ileum at a rate of 8–12 bpm, finally arriving at the jejunum (Christensen et al., 1966). In the colon, slow waves are generated and conducted throughout its length (Smith et al., 1987). ICC located within the muscle layers (ICC-IM) have additional physiological functions. These cells serve as a target for innervation, influencing GI motility in response to neural inputs (Beckett et al., 2003, 2005; Powley et al., 2008), set the resting membrane potential of smooth muscle cells by releasing the hyperpolarizing gasotransmitter, carbon monoxide (Farrugia et al., 2003; Sha et al., 2007) and mechanoreception (Won et al., 2005).

## Pacemaker activity

Pacemaker activity in the heart produces rhythmic atrial and ventricular contractions. By contrast, GI motility in the baseline is regulated by slow wave activity generated by the ICC network, but significant contractions are regulated by a complex interplay between neurogenic and myogenic factors locally, and endocrine signals systemically (Cheung and Wu, 2013). The GI tract produces two types of motion: peristalsis, which are rhythmic contractions that propel intraluminal contents and encourage mixing, and segmentation, which are ring contractions that divide the intraluminal contents but do not produce net movement along the GI tract (Weisbrodt, 1981; Table 1). In the human heart, the normal rate of the discharge of the SA node is between 60 and 100 beats per min (bpm). Subsidiary pacemakers discharge at slower rates and are normally reset by the SA node. These include the AV node, which discharges at 40–60 bpm, and the Purkinje system that discharges at 20–40 bpm. In the GI tract, ICC-MY at the greater curvature of the stomach discharges with a rate of 5–8 bpm with a rate of 5–8 bpm (Kelly and Code, 1971; Rhee et al., 2011; Cheng, 2015). Thus, ICC are organized into a continuous network, such that most regions of the smooth muscle in the small intestine are capable of generating pacemaker activity. This network runs circumferentially and longitudinally within the tunica muscularis of the GI tract. For the intestines, the dominant pacemaker with the highest rate of discharge is the duodenum, discharging at 12 bpm, with a decreasing frequency along the length to 8 bpm in the ileum (Christensen et al., 1966; Diamant and Bortoff, 1969; Szurszewski et al., 1970). There is a conduction delay between proximal and distal sites, ensuring coordinated contraction in this sequence for moving the luminal contents along the GI tract. Loss of dominant pacemaker will result in the takeover of subsidiary pacemakers in both the heart and GI tract (Homma et al., 2004; Tse, 2015). In the GI tract, three distinct population of cells are found: smooth muscle, ICC, PDGFR-positive cells, which together constitute an integrated united called the SIP syncytium (Sanders et al., 2006). It remains to be elucidated whether smooth muscle or PDGFR-positive cells are capable of taking over pacemaker activity in many GI motility disorders where ICC are lost or absent.

**Table 1:** **Conditions caused by abnormal gastrointestinal electrophysiology and their proposed mechanisms**.

**Condition**	**Electrophysiological Basis**	**References**
Gastroesophageal reflux disease	ICC loss	Shafik et al., 2005
Achalasia	ICC loss	Khelif et al., 2003; Chen et al., 2013
Gastroparesis	ICC loss	O'Grady et al., 2012
Pyloric stenosis	ICC loss; abnormal excitation of the gastric wall muscle; abnormal propagation through the pyloric muscle	Watanuki et al., 1969; Langer et al., 1995; Vanderwinden et al., 1996
Functional dyspepsia	Na^+^ channel or other ion channel dysfunction	Jung et al., 2012
Idiopathic rapid gastric emptying	? Ion channel dysfunction	Bharucha et al., 2011
Unexplained nausea and vomiting	? Ion channel dysfunction; recurrent arrhythmias of abnormal wave propagation and higher wave frequency in the distal stomach	Abell et al., 2009
Mesenteric ischaemia	Ischaemia causes intestinal arrhythmia	Seidel et al., 1999a,b Irimia and Wikswo, 2008
Functional diarrhea	? ICC over activity; ? ion channel dysfunction	Dellon and Ringel, 2006
Function constipation	ICC loss; ? ion channel dysfunction	Camilleri, 2011
Irritable bowel syndrome	ICC loss; altered ICC network; electrophysiological remodeling; Na^+^ channel mutations	Saito et al., 2009; Tana et al., 2010; Eshraghian and Eshraghian, 2011
		Tana et al., 2010
Hirschsprung disease	ICC loss	Yamataka et al., 1995
Chronic pseudo-obstruction	ICC loss; altered ICC network	Feldstein et al., 2003; Struijs et al., 2008
Slow transit constipation	ICC loss	Lyford et al., 2002
Colonic hypomotility associated with anorectal malformations	Abnormal ICC	Kenny et al., 1998

*That non-electrophysiological mechanisms are not included here*.

Molecular mechanisms underlying cardiac and GI pacemaker activity involve both voltage- and Ca^2+^-dependent processes (Lakatta et al., 2006; Takaki et al., 2010). In the heart, the voltage-dependent mechanism involves the funny current (*I*_f_) (Baruscotti et al., 2005) that has several unusual characteristics (DiFrancesco, 1993), including activation by hyperpolarization, permeability to both Na^+^ and K^+^ ions with a small single channel conductance, and modulation by cAMP. The Ca^2+^ mechanism involves spontaneous Ca^2+^ release from the endoplasmic reticulum (ER) (Vinogradova et al., 2005), activating the Na^+^−Ca^2+^ exchanger (*I*_NCX_). Some have contended that the one mechanism is more important, entraining the other (Lakatta and DiFrancesco, 2009). However, numerical studies suggest that both voltage- and Ca^2+^ mechanisms are synergistically coupled to each other, called the coupled clock theory (Maltsev and Lakatta, 2013), a notion that is supported by experimental studies (Yaniv et al., 2014). Interestingly, transient receptor potential channels and store-operated Ca^2+^ entry have been demonstrated in SA node cells, suggesting that these channels may play a role in modulating pacemaker activity in the heart (Ju et al., 2007).

By contrast, ICC-MY are the pacemaker cells of the GI tract (Lees-Green et al., 2011). The maximum diastolic potential is set mainly by the K^+^ channel called ether-a-go-go-related (ERG) channel (Huizinga et al., 2004). Ca^2+^ release from inositol 1,4,5-trisphosphate (IP_3_) receptor-operated stores is followed by Ca^2+^ stimulated uptake by the mitochondria and back to the ER (Suzuki et al., 2000; Ward et al., 2000, 2003). Depletion of intracellular Ca^2+^ activates the Ca^2+^-inhibited, non-selective cationic conductance (Koh et al., 2002). The identity of this channel may be the transient receptor potential canonical 4 or related channels (Walker et al., 2002; Jin et al., 2009). Other currents, such as the voltage-independent, dihydropyridine-insensitive Ca^2+^ conductances and Ca^2+^-activated Cl^−^ conductance (called Ano1) (Gomez-Pinilla et al., 2009; Zhu et al., 2009), may also contribute to pacemaker activity (Lee et al., 2007). This pacemaker current depolarization then activates the T-type Ca^2+^ channels (Lee et al., 2007). Furthermore, experimental evidence suggests that tetrodotoxin-resistant Na^+^ channels play a role in slow wave generation, at least in humans (Strege et al., 2003). The Na^+^ channels found in ICC are encoded by SCN5A, bearing similar electrophysiological properties to the cardiac isoform. Interestingly, these channels are likely to have a role in setting the resting membrane potential and modulate the rate of upstroke and frequency of the slow waves, rather than directing contributing to the pacemaker current *per se* (Strege et al., 2003). The repolarization phase of the slow wave involves several currents, mediated by Ano1 described above (Zhu et al., 2009) and K^+^ currents mediated by the ERG (McKay et al., 2006), Big K^+^ (Zhu and Huizinga, 2008), Ca^2+^-activated K^+^ (Fujita et al., 2001), and rectifier channels (Hatton et al., 2001; Huizinga et al., 2004).

## Ionic contributions of the cardiac action potential and GI slow wave

**Figure 2:**
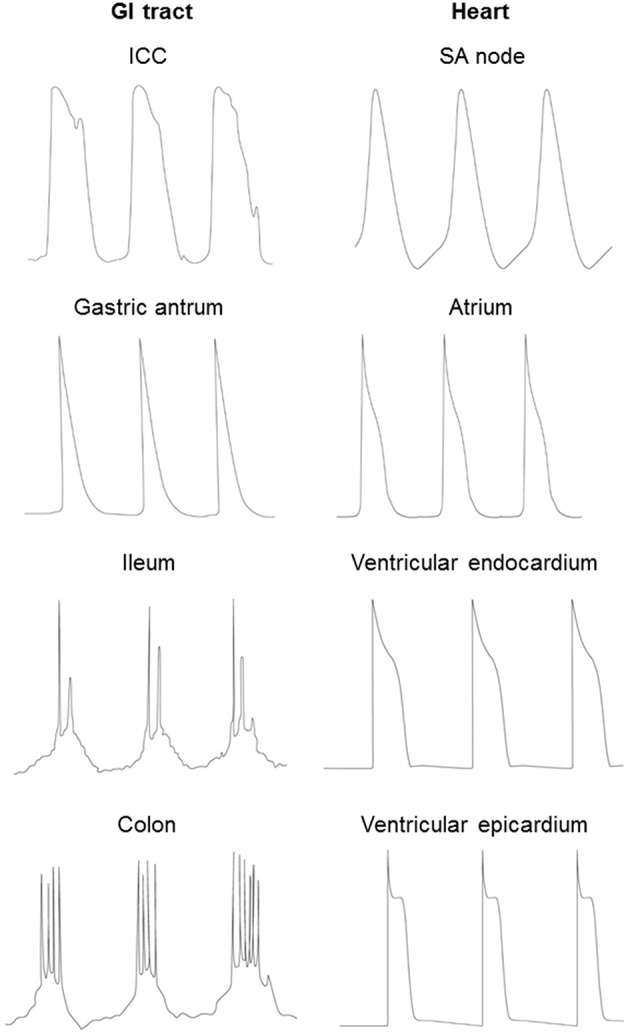
**Different waveforms of the cardiac AP and GI slow wave in different cell types**.

Both the cardiac AP and the GI slow wave result from the sequential opening and closing of ion channels that are located in the plasma membrane (Roden et al., 2002). In both systems, differences in the expression and properties of ion channels are responsible for the heterogeneities in signal waveforms found in different cell types and ensures its normal unidirectional spread through the respective conduction pathways (Figure 2; Nerbonne and Guo, 2002; van Helden et al., 2010; Tse and Yeo, 2015). In the heart, the different cell types are pacemaker cells (sinoatrial and atrioventricular nodes) and cardiomyocytes (epicardium, myocardium and endocardium). In the GI tract, three distinct population of cells are found: smooth muscle, ICC, PDGFR-positive cells, as discussed above (Sanders et al., 2006).

Generation of the APs or slow waves is dependent upon voltage-gated conductances, and their durations are determined by the balance between inward and outward currents (Figure 2). APs of cardiomyocytes have a fast upstroke followed by a spike and plateau morphology, and then delayed repolarization back to the resting membrane potential. Nodal cells have a pacemaker current leading to spontaneous depolarization before the upstroke of the AP.By contrast, in the GI tract, electric activity produced by a gastric antral cell has a triangular morphology, with a fast upstroke followed by rapid repolarization. Smooth muscle in small intestine and colon generate slow waves, which have two phases: an initial depolarizing phase, the pacemaker potential, generated by the ICC-MY (Dickens et al., 1999). The second phase is produced by ICC-IM within the smooth muscle (Dickens et al., 2001). Some cells are capable of producing regenerative Ca^2+^-mediated spikes superimposed upon the slow wave (Suzuki and Hirst, 1999; Lammers and Slack, 2001). The durations of both cardiac APs and GI slow waves can decrease with increasing pacing rates. In cardiac tissue, this was originally described by the restitution hypothesis, which related AP duration to the previous diastolic interval (DI) (Nolasco and Dahlen, 1968). Increased restitution gradient can lead to the formation of APD alternans, wave break and ventricular arrhythmias (Hsieh et al., 2009, 2015; Tse et al., 2016c,e). However, in the GI smooth muscle cells, the restitution curve is flat (Aliev et al., 2000) and therefore breaks in the slow wave conduction are unlikely to be attributed to steep APD restitution.

Conduction of the electrical excitation through the cardiac myocardium and along the GI tract involve mechanisms common to both systems, but additional differences are observed (Saez et al., 2003; van Helden et al., 2010; Veeraraghavan et al., 2014d, 2015; Tse and Yeo, 2015). In the heart, action potential conduction was originally described by the cable theory, positing that the myocardium functions as a syncytium coupled by resistive pathways with capacitances due to the phospholipid bilayer of the cell membranes (Weidmann, 1952). The related core conductor equation makes no assumptions on the structure of the cell membrane. Subsequent experiments revealed that the myocardium consisted of individual cardiomyocytes, which are coupled to each other via nexuses termed gap junctions (Dewey and Barr, 1962; Barr et al., 1965). The latter are hexagonal proteins originally described in the Mauthner cell synapses in goldfish brains (Robertson, 1963). They are non-specific pores that allow the spread of ions and molecules up to 1 kDa in molecular mass (Harris, 2001; Weber et al., 2004). Each gap junction consists of two hemi-channels termed connexons, with each connexon made of six connexin (Cx) subunits. Over 20 Cx isoforms have been identified thus far. In cardiac tissue, Cx43 is expressed in the atria and ventricles (Koval et al., 2014). Cx40 are expressed in the atria and His-Purkinje system and responsible for increasing electrical coupling and CV of the APs in this area (Schrickel et al., 2009). Cx45 alone is sufficient for maintaining electrical conduction through the atrioventricular node, and is also found in the ventricles (Schrickel et al., 2009). Cx30.2 has the lowest unitary conductance out of the cardiac connexins, and its expression in atrioventricular node is responsible for decreasing the CV of AP propagation through this node (Kreuzberg et al., 2006). Other isoforms of Cx, such as 30, 37, and 46 are also found throughout the heart, the reader is referred to this article here for additional information on their respective functions (Verheule and Kaese, 2013). Recent studies have proposed the role of ephaptic coupling in mediating cardiac conduction (Rhett and Gourdie, 2012; Lin and Keener, 2013; Rhett et al., 2013; Veeraraghavan et al., 2014d, 2015, 2014b,c; George et al., 2015).

In the GI tract, two schools of thought were proposed to describe the conduction of slow waves through the GI tract, the core conductor and the coupled oscillator theories (Publicover and Sanders, 1989; Daniel et al., 1994). Like in the heart, the core conductor theory was used to describe electrical propagation along the GI tract, but this theory alone does not fully explain the differing waveforms and frequencies as well as periods of quiescence observed for slow wave propagation. These can be explained by the oscillator model, a clock governs the frequency and a transformer determines the morphology of the waveform (Bardakjian and Diamant, 1994). Two popular models are the Hodgkin-Huxley and Fitzhugh-Nagumo oscillators, incorporating features of both oscillator and conductor theories (Aliev et al., 2000). They accurately describing the physiology of slow wave propagation: changes in frequency along the GI tract, synchronization in short distances and desynchronization over long distances (Aliev et al., 2000; Lin et al., 2006). Recent mapping experiments demonstrated that the pyloric junction is the dominant pacemaker, initiating slow waves that propagate through the small intestine, with decreasing CV and spontaneous conduction blocks along its length (Lammers and Stephen, 2008). Gap junctions may be responsible for facilitating the spread of electrical activity within the ICC network and between different cell types (Daniel et al., 1998; Daniel and Wang, 1999; Seki and Komuro, 2001; Cousins et al., 2003; Daniel, 2004; Hanani et al., 2005). They regulate many aspects of GI physiology, including motility, stomach acid secretion, mucosal barrier function and mediate oral tolerance by antigen transfer (Iino et al., 2001; Ey et al., 2009; Fukushi et al., 2014; Mazzini et al., 2014). Thus, Cx26, 32, and 43 have been found in the stomach, predominantly in the antrum and greater curvature, but rarely in the fundus and pylorus (Maes et al., 2015). Experiments in mouse intestine showed that they may not be necessary for conduction of pacing activity from the ICC to circular smooth muscle (Cho and Daniel, 2005; Daniel et al., 2007). However, gap junction blocker carbonaloxone did decrease the amplitude, frequency and velocity of circular smooth muscle contraction and within the circular smooth muscle network (Schultz et al., 2003), suggesting a modulatory role of gap junctions. However, another blocker, heptanol, did not affect these parameters (Parsons and Huizinga, 2015). Other experiments have shown that gap junctions are not needed for electrical coupling, but instead an electric field mechanism, i.e., ephaptic coupling, is sufficient (Vigmond and Bardakjian, 1995; Sperelakis and McConnell, 2002). It is thus likely that gap junctions play a diminished role in electrical conduction through the GI tract and play more important roles in metabolic regulation, as opposed to electrical coupling in cardiac tissue. Moreover, anisotropic conduction is more important in the heart compared to the GI tract (Spach, 1999; Lammers et al., 2002). In cardiac tissue, CV is much more rapid in the longitudinal compared to the transverse direction. In the gut, the presence of an ICC network means that propagation does not differ significantly in the longitudinal and circumferential directions (Huizinga et al., 1995). In one study, CV was more rapid in the circular compared to longitudinal direction (Lammers et al., 2002), although no significant anisotropy was observed in a different study (Gao et al., 2013). Exacerbations in anisotropic conduction can lead to arrhythmias in both the heart and the GI tract (Allessie et al., 1989; Angeli et al., 2013).

Electrical excitation of cardiac muscle and GI smooth muscle cells induce mechanical contractions via excitation-contraction coupling. In the heart, activation of cardiac muscle elicits an AP, during which Ca^2+^ entry through L-type Ca^2+^ channels provides the necessary current for transverse tubular depolarization and subsequent Ca^2+^-induced Ca^2+^ release from the sarcoplasmic reticulum via the ryanodine receptors (Ozaki et al., 1991). By contrast, phasic contractions in the GI tract involve coordination within the SIP syncytium (Sanders et al., 2014). GI smooth muscle cells receive direct depolarizing current from the ICC for L-type Ca^2+^ channel activation. Its distension also causes depolarization by stretch-sensitive ion channels (Bülbring, 1955; Thorneloe and Nelson, 2005; Kraichely and Farrugia, 2007). Moreover, the migrating myoelectric complex is a band of excitation that travels slowly across the stomach and intestine, and within this band, slow wave-driven peristalsis occurs (Hall et al., 1982; Sarna et al., 1983; Siegle and Ehrlein, 1987).

## Studying cardiac and GI electrophysiology in humans and animal models

In clinical practice, it is possible to record electrical activity of the heart and the stomach from the skin surface non-invasively using electrocardiography and electrogastrography. Magnetic resonance imaging is an excellent non-invasive method for characterization of structural and metabolic abnormalities in both the cardiovascular and gastrointestinal systems (Leung et al., 2004; Vassiliou et al., 2014; Chan et al., 2015; Tse et al., 2015a,b). Recent developments have focused on the measurement of magnetic signals for studying electrical characteristics in these organs. Thus, magnetocardiography can be used to diagnose and predict the risk of cardiac arrhythmias (Steinhoff et al., 2004; Sato et al., 2012; Kwong et al., 2013; Ito et al., 2014; Yoshida et al., 2015). Similarly, magnetogastrography can be used to study the electrophysiological basis of GI motility disorders (Bradshaw et al., 2016).

Many physiological findings described above have been derived from experiments conducted in animal models, but their limitations must be recognized. Significant differences in physiology are observed between different species, largely due to isometric scaling. For example, heart rate in humans are between 60 and 100 bpm but occurs at 600 bpm in mice (Tse et al., 2016c). Similarly, for the gastrointestinal tract, peristalsis occurs at a frequency of 8–12 bpm in humans but 30–40 bpm in mice (Christensen et al., 1966; Huizinga et al., 1995). There are also differences in the cellular electrophysiology. Thus, atrial and ventricular APs in mouse hearts have a triangular morphology without the characteristic plateau phase seen in humans and other species such as guinea pigs (Nerbonne and Kass, 2005; Tse et al., 2012, 2016c,d,i; Osadchii, 2014a,b, 2016). In the GI tract, the morphology of gastric slow waves between mouse and humans is largely similar, and in the small intestine and colon, slow waves with superimposed spikes are observed in both species (Sanders et al., 2014), meaning that results from mouse studies are highly translatable to human GI electrophysiology. Animal models are useful as a variety of experimental recording techniques can be used. Thus, cardiac electrical recordings can be obtained from single cells using microelectrode techniques intracellularly (Sano et al., 1959; Allessie et al., 1976), or from the intact organ by extracellular recording techniques such as the monophasic action potential or bipolar electrogram methods (Vigmond and Leon, 1999; Vigmond, 2005; Vigmond et al., 2009; Tse et al., 2016h), which are techniques used routinely in cardiac electrophysiological studies (Yoshida et al., 2012). Optical mapping can provide high resolution of electrical activation patterns and used to determine depolarization or repolarization abnormalities locally in both organ systems (Hsieh et al., 2009; O'Grady et al., 2012; Angeli et al., 2013).

## Clinical relevance: Motility disorders and targets for future therapy

A number of diseases can arise from disturbances in GI electrophysiology, which is summarized in Table 1. Starting from the top of the GI tract, gastroesophageal reflux disease occurs when the reflux of gastric contents causes troublesome symptoms and has been associated with a loss of ICC in the esophagogastric junction (Shafik et al., 2005). Achalasia, defined as absence of esophageal peristalsis and impaired relaxation of the lower esophageal sphincter, has been attributed to a loss of ICC-IM in the lower esophagus (Chen et al., 2013). Allgrove syndrome is a rare autosomal recessive disorder characterized by a triad of achalasia, alacrima and Addsonian features. The underlying pathology involves lymphocytic infiltration of myenteric plexus and may involve loss of ICC-MY or ICC-IM (Khelif et al., 2003). Gastroparesis, i.e., paralysis of the stomach muscle, is characterized by delayed emptying and caused by abnormal slow wave initiation secondary to ICC-MY loss (O'Grady et al., 2012). Pyloric stenosis, which affects infants, is characterized by hyperplasia and hypertrophy of the pyloric muscle. As far back as 1969, it was suggested that the “hypertrophy” of the pylorus may be caused by spasm of the pyloric muscle, which was associated with abnormal excitation of the gastric wall muscle or disturbance of propagation in the pyloric portion (Watanuki et al., 1969). Reduced number of ICC has also been implicated in this disorder (Langer et al., 1995; Vanderwinden et al., 1996). Functional dyspepsia is persistent epigastric pain and fullness and early satiety without an organic cause. Some proposed mechanisms are abnormal gastric motor function, altered visceral sensitivity and Helicobacter pylori infection. Loss-of-function in the SCN5A gene encoding for the α-subunit of the Na^+^ channel is known to cause Brugada syndrome, a primarily right ventricular arrhythmogenic disorder (Brugada and Brugada, 1992). A recent study of patients with either Brugada syndrome or functional dyspepsia revealed that some Brugada patients with SCN5A mutations have functional dyspepsia (Jung et al., 2012). Thus, the role of abnormalities in Na^+^ and other channels in functional dyspepsia awaits further clarification.

By contrast, idiopathic rapid gastric emptying could involve increased gastric contractility or reduced resistance to gastric outflow at the pylorus (Bharucha et al., 2011). Its mechanism is uncertain but could conceivably involve ion channel dysfunction, such as stretch-activated channels involved in mechanosensation or any of the ion channels mediating gastric slow wave initiation and propagation. In unexplained nausea and vomiting, both neuropathic changes and abnormal gastric electrophysiology have been detected (Abell et al., 2009). Gastric serosal electrophysiological study using serosal electrogastrographic recordings demonstrated recurrent arrhythmias of abnormal wave propagation and higher frequency in the distal stomach. The precise electrophysiological mechanism of arrhythmia, such as focal activity or reentry, will need to be elucidated. Like idiopathic rapid gastric emptying and unexplained nausea and vomiting, arrhythmogenesis is also observed in mesenteric ischaemia, but this is a consequence of rather than the cause (Seidel et al., 1999b). The problem of this condition is that it has a high mortality, due to the disease process itself and the fact that diagnosis is often delayed because of non-specific symptoms. Thus, there is a need of developing means to detect ischaemia early. A highly sensitive magnetometer, such as superconducting quantum interference device (SQUID), could detect a decrease in basic electrical rhythm frequency, which is suggestive of ischaemia (Seidel et al., 1999a). Indeed, a recent study used magnetoenterographic imaging with a SQUID biomagnetometer to measure spatiotemporal return map in pigs. Before induction of ischemia, no intestinal arrhythmias were observed, and the spatiotemporal return map perimeter was relatively constant in time. However, after mesenteric artery ligation, arrhythmias were detected, and associated with spatiotemporal return map perimeter showing statistically significant variations in information dimensionality (Irimia and Wikswo, 2008). However, the need to use a magnetically shielded room and high costs have prohibited the use of SQUID in clinical practice.

IBS is a chronic relapsing and remitting functional disorder of the GI tract. It consists of a triad of altered bowel habits, bloating, and abdominal pain without an organic cause (Sinagra et al., 2016). It can take on a diarrhea- or constipation- predominant phenotype, which must be distinguished from functional diarrhea (Dellon and Ringel, 2006) or constipation, respectively (Dellon and Ringel, 2006; Camilleri, 2011; Cheung et al., 2013). Loss of ICC has been suggested as a pathophysiological mechanism underlying this condition (Eshraghian and Eshraghian, 2011). Mutations in Na^+^ channels have been implicated in IBS (Saito et al., 2009). There is increasing evidence that altered microbiota profile in the intestines may be responsible for the symptoms of irritable bowel syndrome (Tana et al., 2010; Ng et al., 2013), and may increase the activity of intestinal Cl^−^ channels (Chang and Talley, 2010). Inflammatory changes in IBS (Der et al., 2000) can alter ICC network and result in electrophysiological remodeling (Akbarali et al., 2010). Hyperexcitability of nociceptive dorsal root ganglia could explain the abdominal pain (Beyak and Vanner, 2005). Finally, the following disorders involve reduced motion of the colon: Hirschsprung disease (Yamataka et al., 1995), chronic pseudo-obstruction (Feldstein et al., 2003; Struijs et al., 2008), slow transit constipation (Lyford et al., 2002) and colonic hypomotility associated with anorectal malformations (Kenny et al., 1998). In each of these condition, loss or abnormal ICCs has been demonstrated.

How can elucidation of the electrophysiological mechanisms underlying GI motility disorders enable the development of better treatment? Ion channels have been targets for anti-arrhythmic therapy in the heart, and have enormous potential to be targeted for the management of GI motility disorders. For example, lubiprostone is an agonist of the chloride channel protein 2, which promotes the Cl^−^ efflux out of intestinal cells into the lumen, for the management of functional constipation or constipation-predominant IBS (Camilleri et al., 2006; Andresen et al., 2007). Crofelemer, by contrast, inhibits both chloride channel protein 2 and also the cystic fibrosis transmembrane regulator channel to reduce Cl^−^ efflux. It has been licensed for HIV-associated diarrhea (Yeo et al., 2013), and has therapeutic potential in conditions such as functional diarrhea or diarrhea-predominant IBS (Manabe et al., 2010). TRPCs, which mediate nociception, may be useful targets for managing abdominal pain symptoms in IBS (Hicks, 2006). Analogous to bradyarrhythmias such as sick sinus syndrome or atrioventricular blocks in the heart, pharmacotherapy or pacemakers can be used to manage GI hypomotility disorders, depending on the disease severity. In gastroparesis, dopamine receptor antagonists with prokinetic effects such as metoclopramide and domperidone, or macrolide antibiotics, can be used. In more severe cases, pacemaker implantation for gastric electrical stimulation is an effective treatment with improvements in gastric emptying rates (Abrahamsson, 2007). Colonic electrical stimulation has demonstrated efficacy of increasing colon transit time in animal studies, and is a potential treatment option for chronic functional constipation or constipation-predominant IBS refractory to medical therapy in humans (Chen S. et al., 2016). For fast rhythms in the heart, anti-tachycardia pacing is a treatment option (Aonuma et al., 1985); whether this could also be useful in gastrointestinal tachyarrhythmias remains to be elucidated.

In conclusion, most GI motility disorders have an electrophysiological basis, and ion channel dysfunction is increasingly recognized as potential causes. GI electrophysiology is a fascinating subject with much to be learnt. A detailed understanding of the complex spatiotemporal dynamics of GI excitation will enable the development of novel therapy for managing GI motility disorders effectively.

## Author contributions

GT design of manuscript; drafted and critically revised the manuscript for important intellectual content; preparation of figures. EL acquired and interpreted primary research papers; critically revised the manuscript for important intellectual content; preparation of figures. VT acquired and interpreted primary research papers; critically revised the manuscript for important intellectual content. JY analyzed and interpreted primary research papers; critically revised the manuscript for important intellectual content. SW drafted and critically revised the manuscript for important intellectual content; preparation of figures. All authors approved the final version, ensured that the text is accurate and agreed to be accountable for all aspects of the work.

### Conflict of interest statement

The authors declare that the research was conducted in the absence of any commercial or financial relationships that could be construed as a potential conflict of interest.
